# Chronic Intermittent Hypoxia Aggravates the Pyroptosis of Renal Tubular Epithelial Cells Through Regulating Mitophagy‐Mediated NLRP3 Inflammasome Activation

**DOI:** 10.1002/jbt.71159

**Published:** 2026-10-29

**Authors:** Yuwei Zhang, Hongyan Tai

**Affiliations:** ^1^ International Medical Department The Second Xiangya Hospital of Central South University Changsha City Hunan Province China; ^2^ Department of Geriatrics The Second Xiangya Hospital of Central South University Changsha City Hunan Province China

**Keywords:** chronic intermittent hypoxia, NLRP3 inflammasome, PINK1‐mediated mitophagy, pyroptosis, renal injury

## Abstract

Obstructive sleep apnea (OSA)‐induced renal injury is triggered by chronic intermittent hypoxia (CIH). Our previous article has revealed that CIH triggers the pyroptosis of renal tubular epithelial cells through the activation of NLRP3 inflammasome, whereas the mechanism by which CIH activates NLRP3 inflammasome remains elusive. Rats and HK‐2 cells were exposed to CIH to establish CIH models. Histopathological staining, blood urea nitrogen (BUN) and serum creatinine (Scr) levels were examined to assess renal injury. Western blot and immunofluorescence assay were used to assess proteins related to NLRP3 inflammasome, pyroptosis and mitophagy. Mitochondrial membrane potential and production of ROS and ATP were examined to evaluate mitochondrial function. CCK‐8, LDH release and ELISA assays were performed to assess cell injury and inflammation. Mitochondrial division inhibitor 1 (Mdivi‐1) was applied for a rescue assay. CIH induced renal injury and suppressed mitophagy in vivo, evidenced by worsening pathological features, elevated Scr and BUN levels, reduced colocalization of Tom20 with LC3B, and downregulated expression of LC3‐II/I ratio, Atg5, PINK1 and Parkin. PINK1 overexpression restored mitophagy by enhancing colocalization of mitochondria with LC3B, upregulating LC3‐II/I ratio and Atg5, and restored mitochondrial function of CIH‐exposed HK‐2 cells through enhancing mitochondrial membrane potential, reducing ROS production and increasing ATP content. PINK1 overexpression alleviated cell viability, inflammation and NLRP3 inflammasome‐mediated pyroptosis in CIH‐exposed HK‐2 cells, which were partly abolished by Mdivi‐1. PINK1 attenuated NLRP3 inflammasome‐mediated pyroptosis in CIH‐exposed HK‐2 cells partially through restoring mitophagy and mitochondrial function, providing potential therapeutic strategies for OSA‐related kidney diseases.

## Graphical abstract

Chronic intermittent hypoxia induced downregulation of PINK1, leading to deficient mitophagy, accumulation of damaged mitochondria and excessive ROS production, which further promoted the NLRP3 inflammasome complex assembly to trigger pyroptosis and the IL‐1β/IL‐18 release in renal tubular epithelial cells.

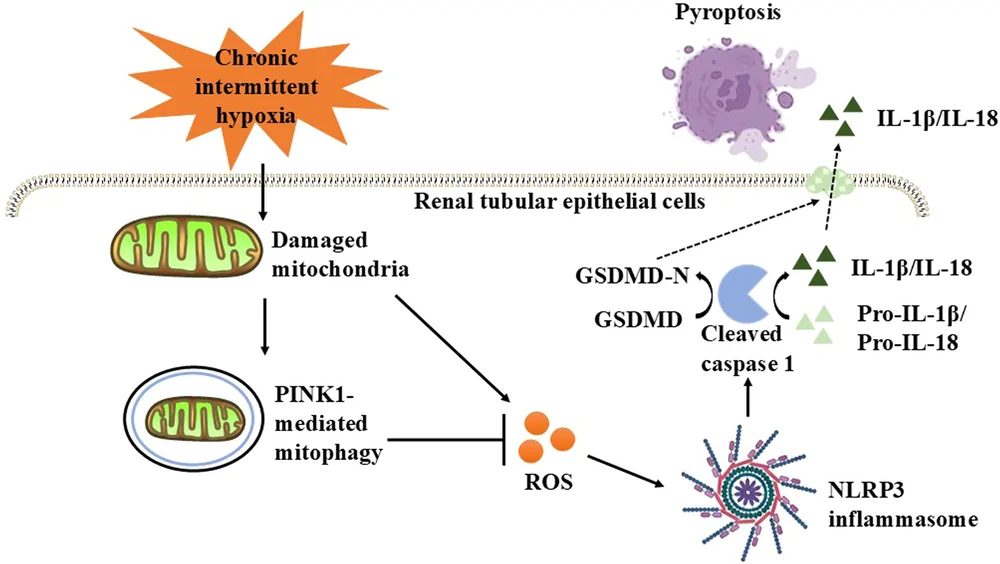

## 1 Introduction

Obstructive sleep apnea (OSA), characterized by recurrent episodes of complete or partial collapse of the upper airway during sleep, is a highly prevalent sleep‐related breathing disorder, affecting 9%–17% of females and 25%–30% of males in the United States [1, 2]. Chronic intermittent hypoxia (CIH), triggered by repetitive upper airway obstruction, is the key driver of OSA pathophysiology [3]. The resultant target organ damage has become a major clinical concern, with frequent complications encompassing cardiovascular dysfunction, cognitive dysfunction, cancer and renal impairment [4, 5, 6, 7]. The kidney, as a high‐oxygen‐demand organ, is highly susceptible to the CIH pattern induced by these cyclic oxygen desaturation‐reoxygenation events. Epidemiological evidence has shown that sleep disorders and chronic kidney disease (CKD) often co‐exist, and CIH exacerbates renal dysfunction and accelerates CKD progression [7, 8, 9]. To date, there is mounting awareness of CIH‐related renal injury, yet the exact cellular and molecular mechanisms driving this process remain poorly defined.

The engagement of the NOD‐like receptor pyrin domain‐containing protein 3 (NLRP3) inflammasome and the ensuing pyroptosis are implicated in the initiation and progression of kidney diseases [10, 11, 12]. The NLRP3 inflammasome is a cytoplasmic immune sensor that responds to cellular stress signals. It is assembled from three core constituents: the Nod‐like receptor (NLR), the caspase‐1 precursor, and the apoptosis‐related spot‐like protein containing a caspase recruitment domain (ASC) [13]. Activation of the NLRP3 inflammasome leads to autocatalytic cleavage of precursor caspase‐1, and the resultant active caspase‐1 then executes proteolytic cleavage of Gasdermin‐D (GSDMD), liberating its N‐terminal pore‐forming domain (GSDMD‐N), which oligomerizes to form transmembrane pores and ultimately facilitates the release of mature IL‐1β and IL‐18, culminating in pyroptosis and aggravating the inflammatory cascades and cell destruction [10, 14, 15]. Within the pathophysiology of OSA, the occurrence of pyroptosis is considered to aggravate multi‐organ dysfunction under CIH conditions, including renal injury [16, 17]. Our previous study revealed significant pathological changes in rat renal tissues following CIH challenge, accompanied by elevated production of IL‐18/IL‐1β and upregulated expression of NLRP3, caspase1 and GSDMD, indicating that CIH activated the NLRP3 inflammasome and triggered pyroptosis in renal tubular epithelial cells [18]. Nevertheless, the mechanism by which CIH activates NLRP3 inflammasome‐related pyroptosis to aggravate renal injury has not been fully elucidated.

Mitochondria represent highly pleomorphic organelles pivotal for preserving cytoplasmic homeostasis. Mitochondrial impairment is closely implicated in CIH‐induced renal injury [19]. Mitophagy, a selective form of autophagy, operates as a critical quality‐control mechanism, recognizing and eliminating compromised mitochondrial elements to sustain cellular health. Defective mitophagy engenders the accrual of dysfunctional mitochondria, consequently leading to excessive production of reactive oxygen species (ROS) and the liberation of mitochondrial danger signals that drive NLRP3 inflammasome activation [20, 21]. The PTEN‐induced kinase 1 (PINK1)/Parkin ubiquitin ligase pathway stands as the most thoroughly elucidated mitophagy signaling cascade [22]. Existing evidence reveals that PINK1/Parkin‐mediated mitophagy alleviates acute kidney injury by reducing ROS and suppressing NLRP3 activation [23]. Under CIH conditions, multiple target organ cells, including microglia, cardiomyocytes, and bronchial epithelial cells, exhibit mitochondrial damage and altered mitophagy [24, 25, 26]. However, the status of mitophagy in renal tubular epithelial cells under CIH conditions and whether impaired mitophagy acts as the upstream trigger for sustained NLRP3 activation remain largely unknown.

Therefore, this study aims to investigate the status of mitophagy in CIH‐induced renal injury and to further clarify the regulatory mechanism of PINK1/Parkin‐mediated mitophagy underlying NLRP3 inflammasome‐related pyroptosis in CIH‐induced renal injury. This research is anticipated to yield novel mechanistic insights into the etiopathogenesis of OSA‐related renal injury and to identify promising avenues for therapeutic intervention.

## 2 Materials and Methods

### 2.1 Animals and CIH Model Establishment

Male Wistar rats (7–9 weeks old), acquired from Topgene Biotechnology (Changsha, China), were kept in a controlled housing environment (12 h light/dark cycle, 22°C ± 2°C, with ad libitum access to standard chow and drinking water). After 1 week of environmental familiarization, the animals were randomized into two experimental groups (six rats in each group): the Control group and the CIH group. The rats in the CIH model were exposed to the intermittent hypoxic chamber from 10:00 AM to 5:00 PM daily, with 8%–12% oxygen concentration and the oxygen concentration gradually returned to 21% in each cycle for 8 consecutive weeks as we previously described [18]. Animals in the Control group were exposed to normoxic air. The project was approved by the Institutional Animal Care and Use Committee of The Second Xiangya Hospital of Central South University.

### 2.2 Assessment of Renal Function

At the end of the experiment, under anesthesia, blood specimens were harvested via the abdominal aortic route. Biochemical indices of renal function, specifically blood urea nitrogen (BUN) and serum creatinine (Scr), were examined adopting their commercial kits (Catalog No. BC4915 and Catalog No. BC1535, respectively, Solarbio, Beijing, China) following the guidelines of the manufacturer.

### 2.3 Histological Analysis

For histological analysis, kidney tissues were immersion‐fixed in neutral‐buffered 4% paraformaldehyde, processed through standard paraffin embedding protocols, and sectioned into 4 μm thickness. Histomorphological analysis was conducted using hematoxylin and eosin (H&E) staining and periodic acid‐Schiff (PAS) staining. Images were observed under a light microscopy (Olympus, Tokyo, Japan).

### 2.4 Immunofluorescence Staining

The sections underwent deparaffinization and rehydration. Antigen retrieval was performed by heating in citrate buffer (pH 6.0) for 10 min, followed by gradual cooling to ambient temperature. After blockage with 5% bovine serum albumin (Boster Biological Technology Co., Ltd., California, USA) for 2 h, sections were exposed to primary antibodies against LC3B (Catalog No. ab192890, Abcam, Cambridge, UK) and Tom20 (Catalog No. ab186735, Abcam) at 4°C overnight, and then incubated with fluorescent secondary antibodies. Nuclear counterstaining was performed using DAPI staining. Representative micrographs were acquired using a confocal fluorescence microscope (Leica, Wetzlar, Germany).

### 2.5 Cell Culture and Treatment

Human renal proximal tubular epithelial cells (HK‐2; Catalog No. CL‐0109; Procell, Wuhan, China) were propagated in Dulbecco's Modified Eagle Medium (DMEM)/F12 containing 10% fetal bovine serum (FBS; Gibco, Grand Island, NY, USA) and 1% penicillin‐streptomycin mixture at 37°C under humidified atmosphere with 5% CO_2_.

For CIH exposure, cells were placed in a computer‐controlled tri‐gas incubator programmed to deliver repetitive 30 min hypoxic (5% O_2_) and normoxic (21% O_2_) phases. Cells in the Control group were maintained under steady‐state normoxia throughout. To inhibit mitophagy, cells were exposed to mitochondrial division inhibitor 1 (Mdivi‐1; 10 μM; Sigma‐Aldrich, St. Louis, MO) for 1 h prior to CIH exposure and maintained throughout the hypoxic period.

To investigate the regulatory role of PINK1, PINK1‐overexpressing vector (OE‐PINK1) and its empty vector control (OE‐NC) were obtained from GeneChem (Shanghai GeneChem Co., Ltd., China). Transfection of OE‐NC or OE‐PINK1 into HK‐2 cells was conducted employing Lipofectamine 3000 reagent (Invitrogen, Carlsbad, CA) per manufacturer's protocols. Cells were harvested 48 h after transfection.

### 2.6 Quantitative Real‐Time PCR (qRT‐PCR)

Extraction of total RNA was executed employing TRIzol reagent (CWBIO, Beijing, China), and the extracted RNA was then subjected to reverse transcription to synthesize complementary DNA using the HiScript II Q RT SuperMix Kit (Vazyme, Nanjing, China). Subsequently, the qRT‐PCR assay was carried out employing the SYBR Green PCR Master Mix (Lifeint, Xiamen, China) on a Real‐Time PCR system (Applied Biosystems 7500; California, USA) per manufacturer's guidelines. The housekeeping gene β‐actin functioned as the internal standard for transcript normalization. The mRNA abundance of genes was determined employing the 2^−ΔΔCt^ method.

### 2.7 Western Blot

The extraction of total proteins was executed employing RIPA lysis buffer (Invitrogen), followed by the determination of protein concentration using the BCA Protein Assay Kit (Beyotime, Shanghai, China). Protein lysates (30 μg per sample) were fractioned by SDS‐PAGE and electroblotted onto PVDF membranes. After blockage with 5% skimmed milk, membranes were subjected to overnight incubation at 4°C with appropriately diluted primary antibodies against PINK1 (Catalog No. 23274‐1‐AP, Proteintech, Wuhan, China), Parkin (Catalog No. 14060‐1‐AP, Proteintech), LC3B (Catalog No. ab192890, Abcam), Atg5 (Catalog No. ab108327, Abcam), p62 (Catalog No. ab109012, Abcam), NLRP3 (Catalog No. ab263899, Abcam), ASC (Catalog No. NBP1‐78977, Novus Biologicals, USA), cleaved caspase‐1 (Catalog No. #4199, CST, Massachusetts, USA), GSDMD‐N (Catalog No. #39754, CST), IL‐1β (Catalog No. #12703, CST), IL‐18 (Catalog No. #67775, CST), and β‐actin (Catalog No. ab8227, Abcam). On subsequent day, membranes underwent exposure to horseradish peroxidase‐conjugated secondary antibody (Catalog No. ab6721, Abcam) for 90 min at ambient temperature. Signals were visualized employing an enhanced chemiluminescence (ECL) kit (PerkinElmer, Waltham, MA, USA).

### 2.8 Measurement of Mitochondrial Membrane Potential

For quantification of mitochondrial membrane potential, HK‐2 cells were assessed employing the JC‐1 assay kit (Catalog No. C2006, Beyotime). Briefly, cells were exposed to JC‐1 working solution at a final concentration of 10 μg/mL and maintained at 37°C for a 20‐min staining interval. After washing with PBS, the fluorescent images were observed under a confocal fluorescence microscope (Leica). Healthy mitochondria with polarized membrane potential exhibit red J‐aggregate fluorescence, whereas depolarized mitochondria show green monomeric fluorescence.

### 2.9 Measurement of ROS and ATP Production

The production of intracellular ROS was quantified employing the fluorescent probe 2',7'‐dichlorodihydrofluorescein diacetate (DCFDA, Catalog No. S0036S, Beyotime) at a working concentration of 5 μM. The staining reaction proceeded for 30 min at 37°C in the absence of light. After incubation, intracellular ROS production was observed under a confocal fluorescence microscope (Leica). Cellular ATP production was quantified employing a firefly luciferase‐based ATP Assay Kit (Catalog No. S0026, Beyotime) conforming to the manufacturer's guidelines.

### 2.10 Cell Viability Assay

Cell viability was assessed using the Cell Counting Kit‐8 (CCK‐8; Catalog No. C0037, Beyotime) per manufacturer's protocol. HK‐2 cells were inoculated at 5 × 10^3^ cells/well in 96‐well plates and exposed to CIH procedure. 24 h later, 10 μL of CCK‐8 reagent was dispensed into each well, followed by maintaining at 37°C for 2 h. Absorbance at 450 nm was quantified spectrophotometrically (Bio‐Rad, Hercules, CA, USA).

### 2.11 Measurement of Lactate Dehydrogenase (LDH) Release

The release of LDH in cell culture supernatants was examined by the LDH release assay Kit with WST‐8 (Catalog No. C0019S, Beyotime) per manufacturer's protocol. Absorbance at 450 nm was quantified spectrophotometrically.

### 2.12 Enzyme‐Linked Immunosorbent Assay (ELISA)

The supernatants from HK‐2 cells were collected and the secreted inflammatory mediators, including IL‐1β and IL‐18, were quantified employing commercial ELISA kits (Catalog No. CSB‐E08053h and Catalog No. CSB‐E07450h, respectively, CUSABIO, Houston, USA) as per manufacturer's instructions.

### 2.13 Statistical Analysis

Quantitative results are expressed as mean ± standard deviation (SD) from biologically independent experiments (*n* ≥ 3). The statistical software GraphPad Prism 9 (La Jolla, CA, USA) was employed for all analyses. Unpaired student's t‐test was applied for single‐factor comparison, while one‐way ANOVA with Tukey's post‐hoc test was applied for multiple comparisons. Statistical significance was declared at *p* < 0.05.

## 3 Results

### 3.1 CIH Induces Renal Injury and Suppresses Mitophagy In Vivo

To understand the impact of CIH on mitophagy, we constructed a CIH rat model. As shown in Figure 1A, CIH‐exposed rats exhibited significant renal tubular injury, characterized by tubular epithelial cell swelling, vacuolar degeneration, and infiltration of inflammatory cells. Consistently, biochemical indices of renal function, including Scr and BUN, were significantly higher in the CIH group than those in the Control group (Figure 1B), indicating impaired renal function in CIH‐exposed rats. Next, in comparison to the Control group, the protein expression of autophagy substrate p62 was greatly increased, while the LC3‐II/I ratio, Atg5 level and the key mitophagy proteins PINK1 and Parkin were significantly decreased in the CIH group (Figure 1C), hinting that the mitophagy was suppressed in CIH rats. In addition, immunofluorescence staining showed reduced colocalization of the mitochondrial outer membrane protein Tom20 with the autophagosome marker LC3B in the kidneys of CIH rats (Figure 1D), further confirming that CIH suppressed renal mitophagy. Taken together, these results indicated that CIH caused renal injury and inhibited PINK1/Parkin‐mediated mitophagy in vivo.

**Figure 1:**
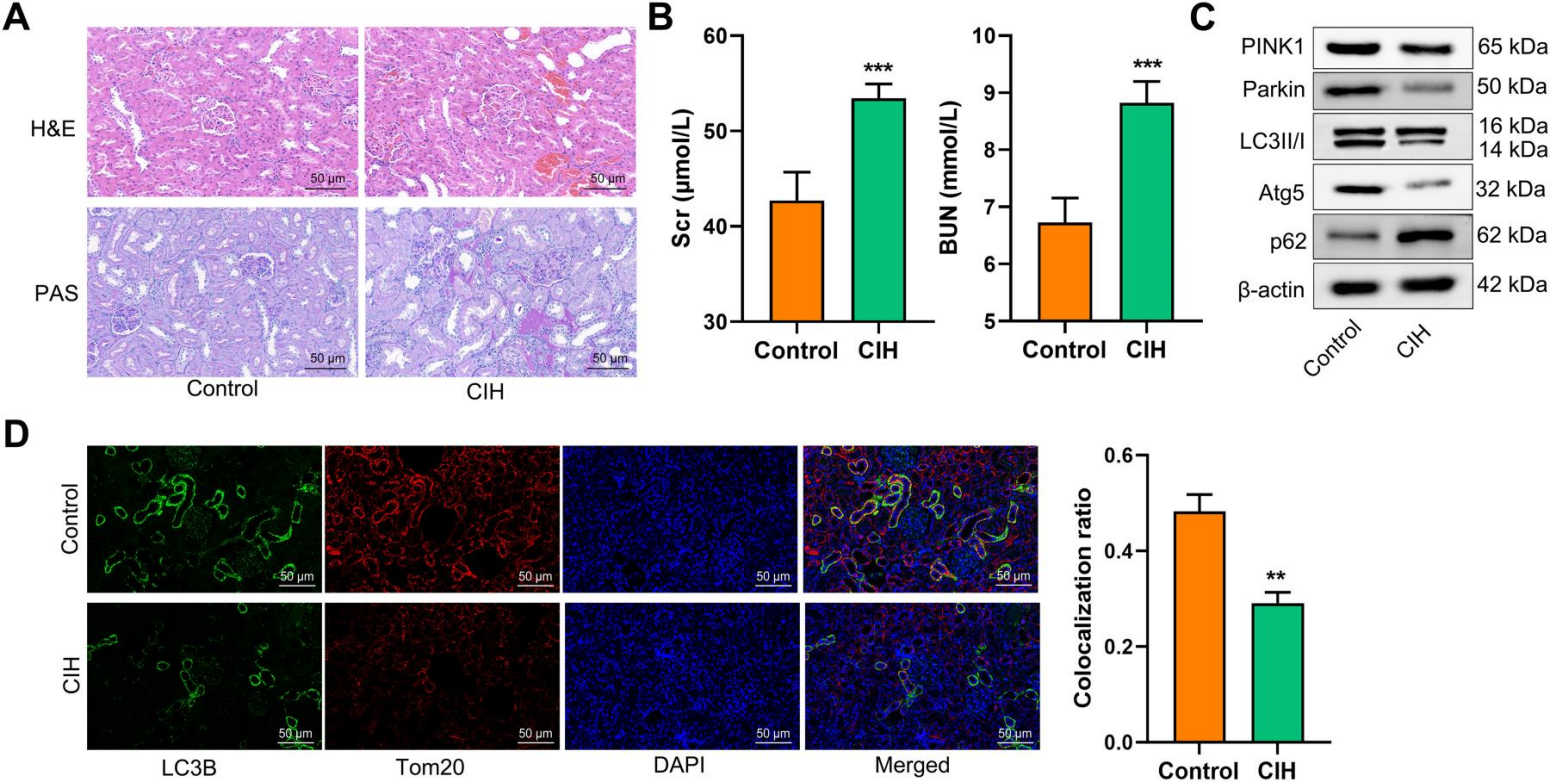
CIH induces renal injury and suppresses mitophagy in vivo. (A) A CIH rat model was constructed, and the representative images of kidney tissues stained by H&E and PAS were presented. (B) Renal function was assessed by Scr and BUN levels. (C) The protein expression of LC3‐II/I, Atg5, p62, PINK1 and Parkin was examined by western blot. (D) Representative immunofluorescence images of colocalization of the mitochondrial outer membrane protein Tom20 with the autophagosome marker LC3B in the kidneys. **p < 0.01, ***p < 0.001.

### 3.2 PINK1 Overexpression Restores Mitophagy and Mitochondrial Function of CIH‐Exposed HK‐2 Cells

Subsequently, we focused on the function and regulation of PINK1/Parkin‐mediated mitophagy in HK‐2 cells exposed to CIH. As shown in Figure 2A, we first introduced OE‐PINK1 vector to HK‐2 cells to achieve PINK1 overexpression, and successful transfection was verified by increased PINK1 mRNA and protein levels. Thereafter, we also found downregulated expression of Parkin, LC3‐II/I and Atg5, accompanied by upregulated expression of p62, in the CIH group in comparison to the Control group, consistent with the in vivo results. However, these alterations were partly abolished by PINK1 overexpression (Figure 2B). Immunofluorescence staining also revealed reduced colocalization of mitochondria (marked by MitoTracker Red) with LC3B in the CIH group in comparison to the Control group, which was partly restored by PINK1 overexpression (Figure 2C,E), confirming that PINK1 overexpression restored mitophagy in CIH‐exposed HK‐2 cells. Furthermore, we assessed mitochondrial function. Compared to the Control group, CIH‐exposed cells exhibited low red immunofluorescence intensity, reflecting a reduction in mitochondrial membrane potential, which was partly restored by PINK1 overexpression (Figure 2D,F), demonstrating that PINK1 overexpression partially reversed CIH‐induced mitochondrial membrane depolarization. Moreover, CIH exposure‐induced excessive ROS production and reduced ATP content in HK‐2 cells were partly attenuated by PINK1 overexpression (Figure 2G,H). These findings demonstrated that PINK1‐mediated mitophagy was essential for maintaining mitochondrial homeostasis of HK‐2 cells under CIH conditions.

**Figure 2:**
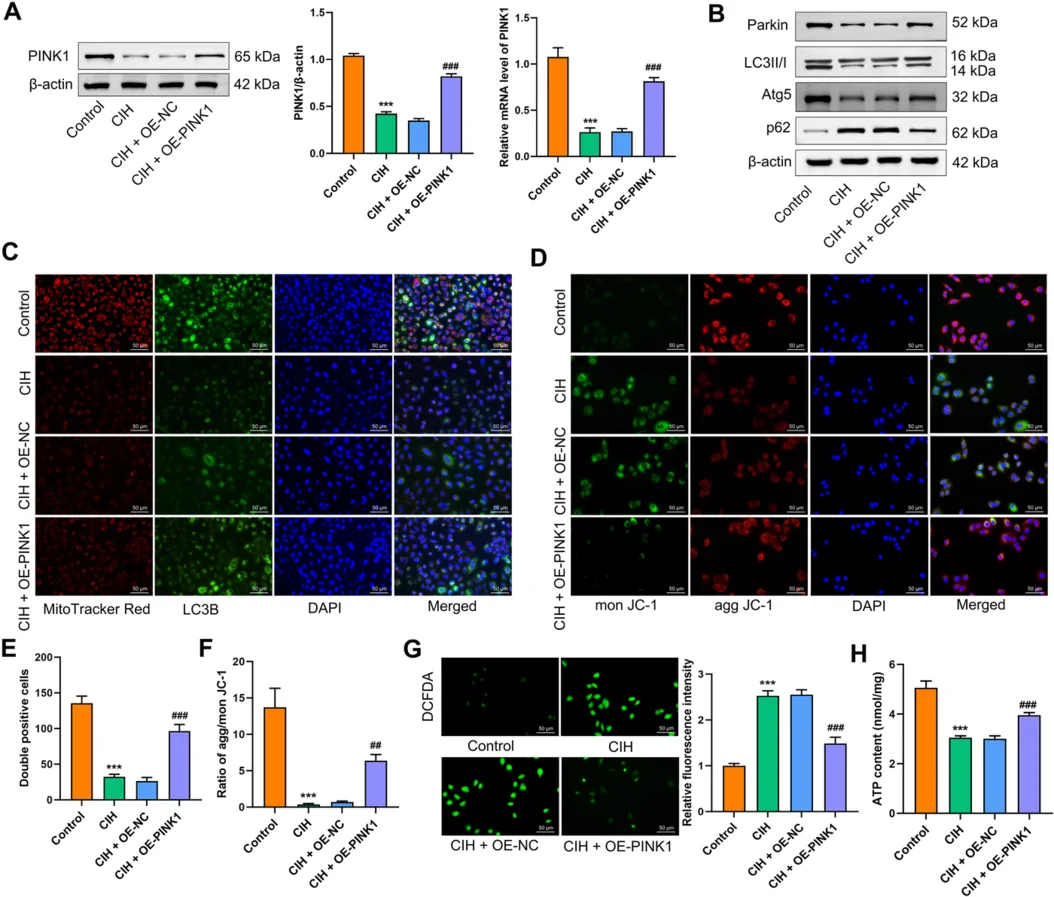
PINK1 overexpression restores mitophagy and mitochondrial function of CIH‐exposed HK‐2 Cells. (A) HK‐2 cells were transfected with OE‐PINK1/OE‐NC, and underwent CIH exposure. The expression level of PINK1 in HK‐2 cells was examined by qRT‐PCR and western blot. (B) The protein expression of LC3‐II/I, Atg5, p62, and Parkin was examined by western blot. (C) Representative immunofluorescence images of colocalization of mitochondria (marked by MitoTracker Red) with LC3B in HK‐2 cells. (D) Mitochondrial membrane potential was assessed by JC‐1 staining. (E) Quantification analysis of cells with LC3B colocalized with mitochondria. (F) Quantification analysis of JC‐1 staining. (G) ROS production was assessed by DCFDA method, and the fluorescence intensity was quantified. (F) ATP production was examined by its commercial kit. ***p < 0.001 vs Control; ^##^ *p* < 0.01, ^###^ *p* < 0.001 vs CIH + OE‐NC.

### 3.3 PINK1 Overexpression Attenuates NLRP3 Inflammasome‐Mediated Pyroptosis in CIH‐Exposed HK‐2 Cells

Next, we investigated the regulatory effect of PINK1‐mediated mitophagy on CIH‐induced pyroptosis in HK‐2 cells. As shown in Figure 3A,B, CIH‐caused cell viability loss and excessive LDH release were notably mitigated by PINK1 overexpression, suggesting that PINK1 overexpression could attenuate cell injury in CIH‐exposed HK‐2 cells. In addition, the elevated protein expression of NLRP3, ASC, cleaved caspase 1, GSDMD‐N, IL‐1β and IL‐18 caused by CIH were markedly reduced following PINK1 overexpression (Figure 3C), revealing that CIH‐activated NLRP3 inflammasome and subsequent pyroptosis were inhibited by PINK1 overexpression, which was further confirmed by the subsequent immunofluorescence staining, which showed that CIH‐enhanced GSMDM‐N expression was weakened by PINK1 overexpression (Figure 3D). Moreover, CIH‐induced elevation in the secretion of IL‐1β and IL‐18 in the culture supernatant was greatly decreased following PINK1 overexpression (Figure 3E). Collectively, these results indicated that PINK1 overexpression protected HK‐2 cells from CIH‐induced pyroptosis.

**Figure 3:**
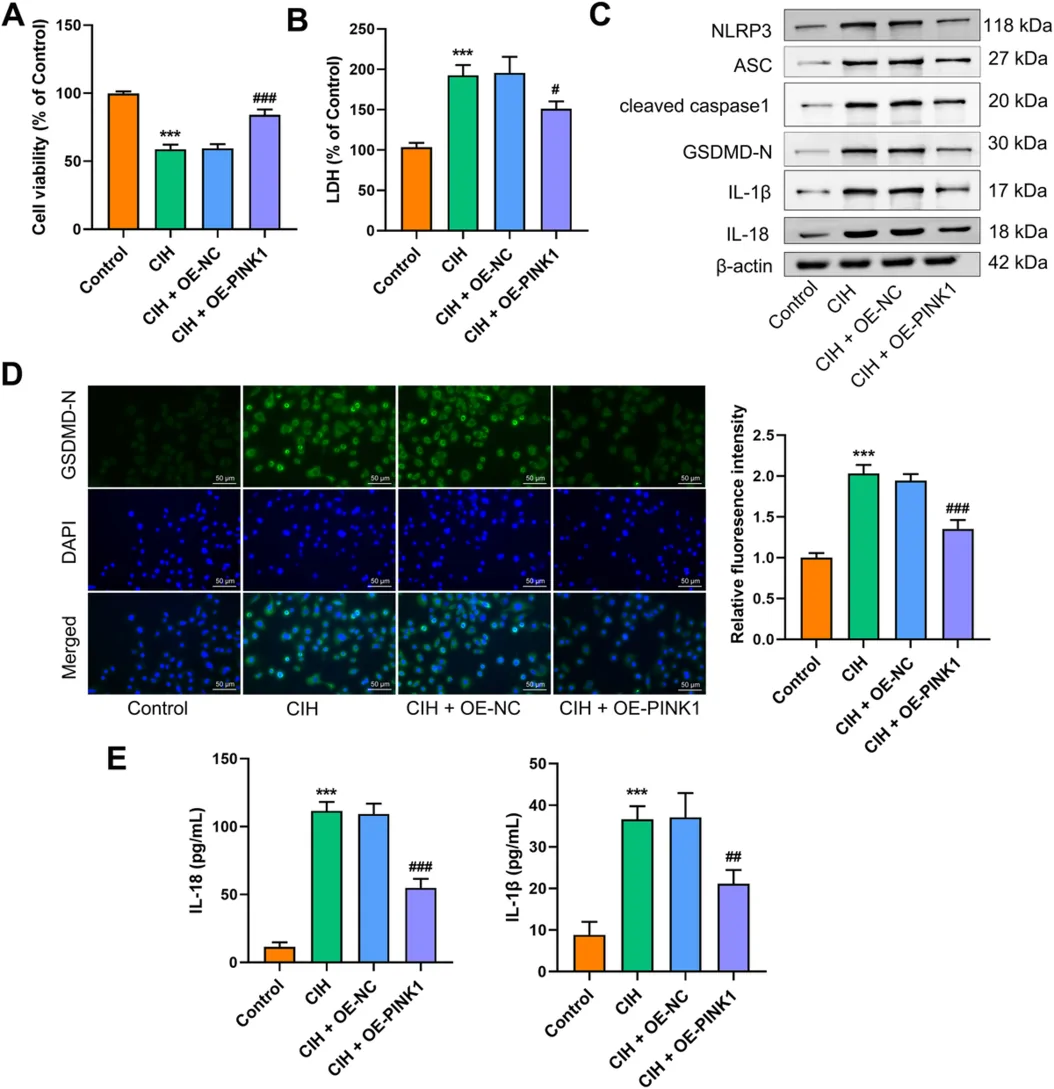
PINK1 overexpression attenuates NLRP3 inflammasome‐mediated pyroptosis in CIH‐exposed HK‐2 cells. (A) HK‐2 cells were transfected with OE‐PINK1/OE‐NC, and underwent CIH exposure. CCK‐8 assay was performed to evaluate cell viability. (B) The LDH release of HK‐2 cells was examined by the LDH Activity Assay kit. (C) The expression of proteins related to NLRP3 inflammasome and pyroptosis was examined by western blot. (D) Representative immunofluorescence images of GSDMD‐N in HK‐2 cells. (E) The release of IL‐1β/IL‐18 was examined by ELISA. ***p < 0.001 vs Control; ^#^ *p* < 0.05, ^##^ *p* < 0.01 and ^###^ *p* < 0.001 vs CIH + OE‐NC.

### 3.4 Inhibition of Mitophagy Abolishes the Protective Role of PINK1 against CIH‐Induced Pyroptosis in HK‐2 Cells

To strengthen the causal relationship between PINK1‐mediated mitophagy and pyroptosis in HK‐2 cells under CIH conditions, we employed Mdivi‐1, a pharmacological inhibitor of mitophagy, for treatment. As expected, Mdivi‐1 treatment alone exacerbated CIH‐induced cell viability loss and LDH release. Notably, Mdivi‐1 greatly abrogated the protective role of PINK1 overexpression against cell viability loss and LDH release (Figure 4A,B). Western blot and immunofluorescence assays showed that under CIH conditions, Mdivi‐1 alone strengthened the activation of NLRP3 inflammasome and subsequent pyroptosis in HK‐2 cells. In contrast, the inhibitory effect of PINK1 overexpression on NLRP3 inflammasome activation and pyroptosis was notably weakened by Mdivi‐1 treatment (Figure 4C,D). Moreover, Mdivi‐1 itself facilitated the production of IL‐1β and IL‐18 of HK‐2 cells exposed to CIH, and meanwhile, the inhibitory effect of PINK1 overexpression on IL‐1β/IL‐18 secretion was reversed by Mdivi‐1 treatment (Figure 4E). Collectively, these findings suggested that PINK1 exerted protective role through mediating mitophagy, and that impaired mitophagy might be a critical upstream event in CIH‐induced NLRP3 inflammasome activation and pyroptosis.

**Figure 4:**
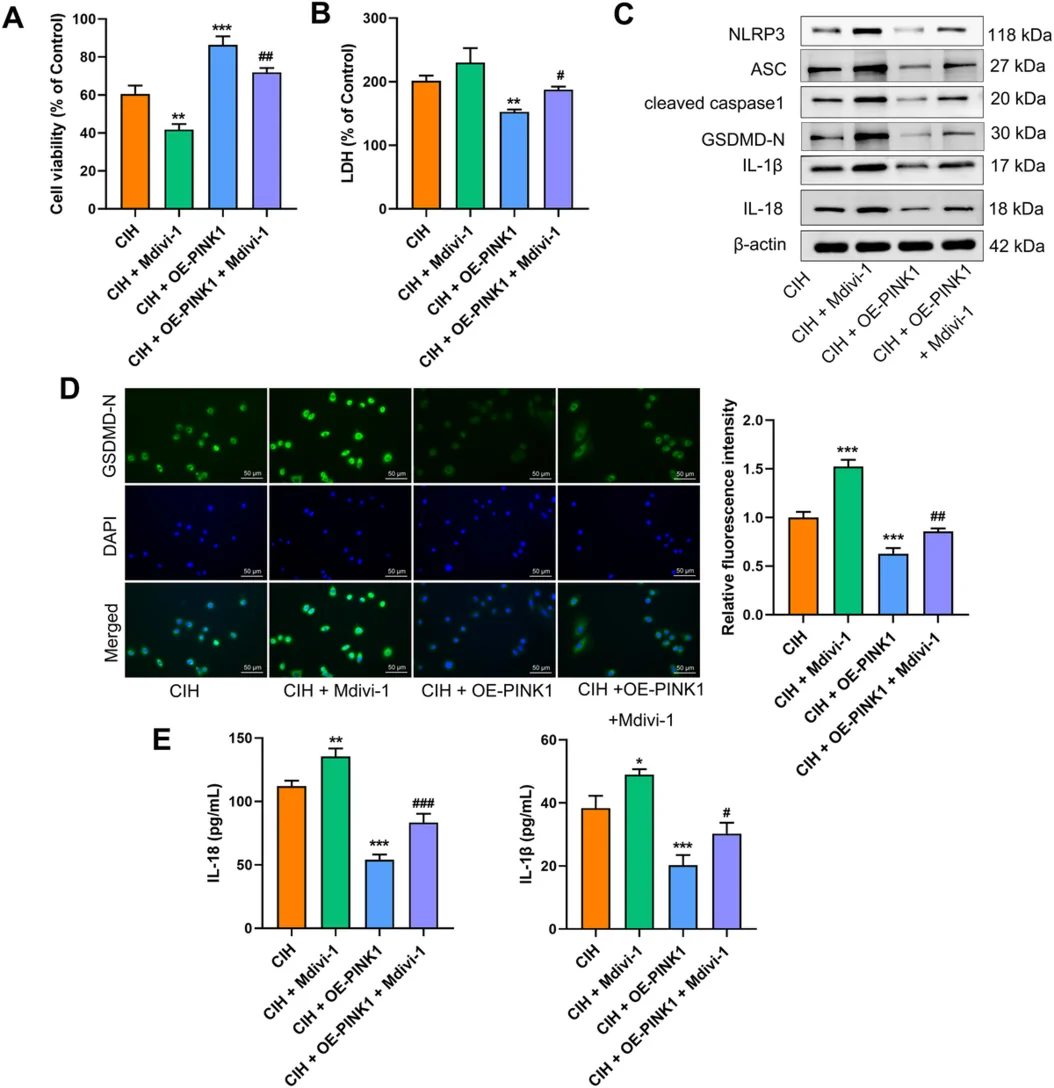
Inhibition of mitophagy abolishes the protective role of PINK1 against CIH‐induced pyroptosis in HK‐2 Cells. (A) HK‐2 cells were transfected with OE‐PINK1, and underwent CIH exposure, with or without Mdivi‐1 treatment. CCK‐8 assay was performed to evaluate cell viability. (B) The LDH release of HK‐2 cells was examined by the LDH Activity Assay kit. (C) The expression of proteins related to NLRP3 inflammasome and pyroptosis was examined by western blot. (D) Representative immunofluorescence images of GSDMD‐N in HK‐2 cells. (E) The release of IL‐1β/IL‐18 was examined by ELISA. *p < 0.05. **p < 0.01 and ***p < 0.001 vs CIH; ^#^ *p* < 0.05, ^##^ *p* < 0.01 and ^###^ *p* < 0.001 vs CIH + OE‐PINK1.

## 4 Discussion

Our results demonstrated the impairment of mitophagy following CIH‐induced renal injury. CIH exposure induced downregulation of PINK1, leading to deficient mitophagy, accumulation of damaged mitochondria and excessive ROS production, which further promoted NLRP3 inflammasome assembly and thereby triggerred pyroptosis and subsequent IL‐1β/IL‐18 release in renal tubular epithelial cells. PINK1 overexpression attenuated NLRP3 inflammasome‐mediated pyroptosis in CIH‐exposed HK‐2 cells by restoring mitophagy and mitochondrial function. Therefore, our findings provide novel insights into the molecular mechanisms of OSA‐related kidney diseases and identify PINK1‐mediated mitophagy as a potential therapeutic target.

Impairment of mitochondrial quality control, especially mitophagy, and mitochondrial dysfunction are well‐established consequences of CIH exposure [27, 28]. Wang et al. [28] disclosed that disruption of mitophagy was correlated with ultrastructural damage of mitochondria and impaired mitochondrial function in CIH‐exposed rats. In addition, inhibited mitophagy, damaged mitochondria, and elevated inflammatory responses and oxidative stress were also observed in bronchial epithelial cells, myocardial cells and neurons following CIH exposure [26, 29, 30]. To date, the precise mechanisms linking CIH to mitochondrial damage and the downstream pathological consequences in renal tubular cells remained unclear. Here, we showed that CIH suppressed mitophagy, accompanied by the accumulation of damaged mitochondria. This was evidenced by reduced colocalization of the mitochondrial marker with the autophagosome marker LC3B, a decreased LC3‐II/I ratio, and elevated p62 levels in both CIH‐exposed rat kidneys and cultured HK‐2 cells. Mitophagy inhibition triggers a cascade of mitochondrial deterioration, including collapse of mitochondrial membrane potential, excessive ROS generation, and ATP exhaustion.

The functional interconnection between the PINK1‐Parkin pathway and NLRP3 inflammasome‐driven pyroptosis has been extensively investigated [31, 32]. Physiologically, PINK1 accumulates on the outer mitochondrial membrane and recruits the E3 ubiquitin ligase Parkin, which then drives the ubiquitination‐dependent engulfment of mitochondria by autophagosomes. Consequently, the PINK1/Parkin pathway is recognized as a principal effector of mitophagy [22]. The NLRP3 inflammasome serves as a critical sensor of mitochondrial danger signals. It has been reported that mitochondrial impairment‐induced apoptotic cascade, coupled with excessive ROS production, can activate the NLRP3 inflammasome, ultimately facilitating pyroptosis [33, 34, 35]. Our results demonstrated that CIH‐induced impairment of PINK1‐mediated mitophagy and mitochondrial dysfunction led to robust NLRP3 inflammasome activation and subsequent pyroptosis, as characterized by increased expression of NLRP3, ASC, cleaved caspase‐1 and GSDMD‐N, accompanied by elevated secretion of IL‐1β and IL‐18. This is consistent with the established paradigm that mitochondrial dysfunction secondary to mitophagy deficiency is a major upstream event in NLRP3 activation.

Currently, PINK1‐mediated mitophagy has emerged as a particularly attractive target for disrupting the CIH‐driven vicious cycle of “mitochondrial damage‐insufficient mitophagy‐amplified inflammation,” and the enhancement of PINK1‐mediated mitophagy may be a promising strategy for eliminating NLRP3 activation signals, attenuating NLRP3 inflammasome activation and subsequent pyroptosis in human diseases. For instance, Li et al. [24] reported that Atraric acid suppressed CIH‐elicited cardiac inflammatory cascades, oxidative stress, and NLRP3 inflammasome activation through enhancing PINK1/Parkin‐mediated mitophagic clearance. Li et al. [36] suggested that fibroblastic reticular cell‐derived exosomes represented a viable strategy for attenuating septic acute kidney injury, as these exosomes could inhibit pyroptosis and improve kidney function by promoting PINK1/Parkin‐mediated mitophagy and concomitant blockade of NLRP3 inflammasome assembly, ultimately conferring improved survival outcomes in sepsis. Furthermore, pharmacological inhibition of STAT3 greatly blocked NLRP3 inflammasome activation by triggering PINK1‐dependent mitophagy [37]. In terms of this article, we substantiated that PINK1 overexpression exerted definitive protective effects against mitochondria dysfunction by restoring mitophagy. Meanwhile, PINK1 overexpression also protected HK‐2 cells against CIH‐induced NLRP3 inflammasome activation and pyroptosis. Furthermore, we employed Mdivi‐1 to pharmacologically inhibit mitophagy in rescue assays. The complete abrogation of PINK1's protective effects by Mdivi‐1 indicated that mitophagy might serve as a critical mediator linking PINK1 to NLRP3 inflammasome suppression and pyroptosis prevention. These findings suggested that strategies aimed at enhancing PINK1‐mediated mitophagy might hold therapeutic potential for OSA‐related kidney diseases.

However, there are several limitations in our study. First, we did not validate the therapeutic efficacy of PINK1‐mediated mitophagy in a CIH animal model; further studies are therefore warranted to perform such in vivo validation. Second, the specific mechanisms by which CIH suppresses PINK1 expression remain incompletely understood. CIH‐activated hypoxia‐inducible factor‐1α and the consequent metabolic reprogramming may transcriptionally repress PINK1 or perturb its upstream regulators, such as adenosine monophosphate‐activated protein kinase (AMPK) and FoxO3a, which govern both mitochondrial biogenesis and PINK1 abundance [24, 27, 38]. Moreover, augmented ubiquitin‐proteasome or autophagy‐lysosomal degradation of PINK1 under CIH conditions could further lower its stability and expression [29]. Hence, elucidating which mechanism predominates in CIH‐exposed renal tubular epithelial cells requires further investigation. Third, we focused primarily on the PINK1/Parkin pathway, whereas other mitophagy mechanisms, such as the BNIP3/NIX pathway, might also play a role in maintaining mitochondrial quality control under CIH conditions [39]. Lastly, Mdivi‐1 is not a direct or specific mitophagy inhibitor. Although our rescue experiments with Mdivi‐1 support a protective role of mitophagy, pharmacological inhibition alone cannot definitively establish mitophagy dependence. Future studies using genetic approaches, such as PINK1 or Parkin knockdown by siRNA, are warranted to validate our findings. Future investigations should address these unresolved issues and explore the translational value of mitophagy‐enhancing strategies in OSA patients with kidney diseases.

## 5 Conclusion

In summary, our findings reveal a novel mechanistic link between CIH and renal injury: CIH aggravates pyroptosis of renal tubular epithelial cells by inhibiting PINK1/Parkin‐mediated mitophagy, leading to accumulation of damaged mitochondria, excessive ROS production and subsequent NLRP3 inflammasome activation. Restoration of PINK1 expression or enhancement of mitophagy may represent promising therapeutic strategies for preventing kidney disease progression in patients with OSA.

## Author Contributions

**Yuwei Zhang:** conceptualization, writing – review and editing, writing – original draft, funding acquisition, data curation, project administration. **Hongyan Tai:** methodology, data curation, formal analysis, software, validation, visualization, writing – original draft, Investigation.

## Conflicts of Interest

The authors declare no conflicts of interest.

## Data Availability

All data analyzed during this study are included in this published study.
